# Rupture non traumatique de la rate révélée par une insuffisance rénale aiguë

**DOI:** 10.11604/pamj.2019.33.176.19562

**Published:** 2019-07-05

**Authors:** Yawovi Mawufemo Tsevi, Eyram Yoan Makafui Amekoudi, Kodjo Abossisso Sakiye, Kwame Doh, Kossi Akomola Sabi, Komi Dzidzonu Nemi, Kodjo Agbeko Djagadou, Eugène Attisso, Clement Kan Ackounddou

**Affiliations:** 1 Service de Néphrologie et d'Hémodialyse, Centre Hospitalier Universitaire Sylvanus Olympio de Lomé, Université de Lomé, Togo; 2 Service de Chirurgie Viscérale, Centre Hospitalier Universitaire Sylvanus Olympio de Lomé, Université de Lomé, Togo; 3 Service d'Anatomo-pathologie, Centre Hospitalier Universitaire Sylvanus Olympio de Lomé, Université de Lomé, Togo; 4 Service de Médecine Interne, Centre Hospitalier Universitaire Sylvanus Olympio de Lomé, Université de Lomé, Togo; 5 Service de Néphrologie, Hémodialyse et de Transplantation Rénale du CHU de Yopougon, Université Felix Houphouet Boigny, Abidjan, Cote d'Ivoire

**Keywords:** Rupture non traumatique de la rate, insuffisance rénale aiguë, toxique traditionnelle, Non-traumatic splenic rupture, acute renal failure, traditional toxic

## Abstract

Nous rapportons dans ce travail un cas d'hématome sous capsulaire spontané rompu de la rate, induite par un toxique traditionnel, et révélé par une insuffisance rénale aigue avec hémolyse intravasculaire.

## Abstract (en)

We report a case of rupture of spontaneous subcapsular splenic hematoma caused by toxic product and revealed by acute renal failure with intravascular hemolysis.

## Introduction

Les ruptures non traumatiques ou spontanées, de la rate (RNTR) sont rares mais peuvent être mortelles [1]. Le diagnostic est souvent difficile. Le retard de la prise en charge thérapeutique, ainsi que la gravité de la pathologie sous-jacente expliquent les taux élevés de cette mortalité [2]. Les RNTR peuvent survenir sur une rate normale ou pathologique. Elles nécessitent dans la majorité des cas une splénectomie [3]. Les causes non traumatiques telles que les maladies infectieuses, les maladies hématologiques et les cancers sont les plus décrites [2,4,5]. Nous rapportons dans ce travail un cas d'hématome sous capsulaire spontané rompu de la rate, induite par un toxique traditionnel, et révélé par une insuffisance rénale aiguë avec hémolyse intravasculaire.

## Patient et observation

**Figure 1:**
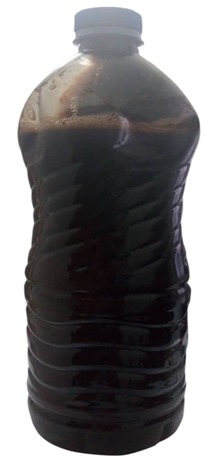
Toxique traditionnelle conditionnée dans un bidon d'Evian

**Figure 2:**
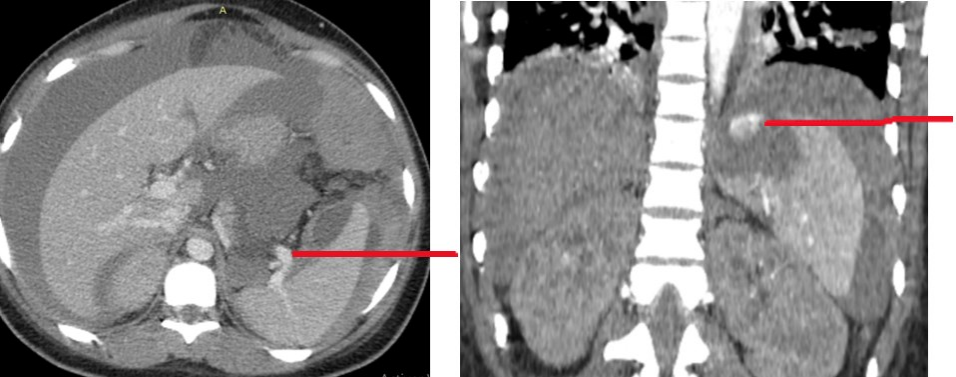
TDM abdominale montrant la rupture splénique

**Figure 3:**
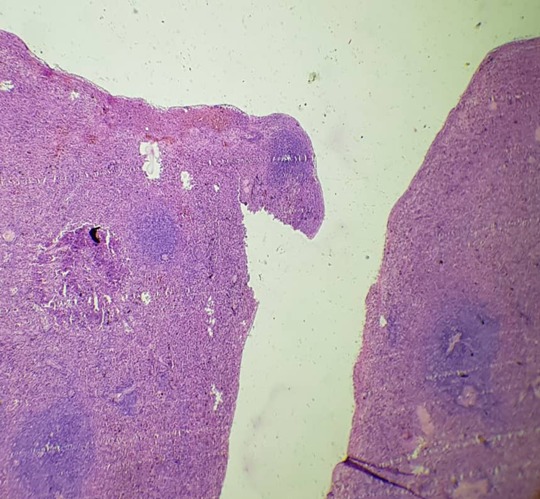
Fracture de la rate

**Figure 4:**
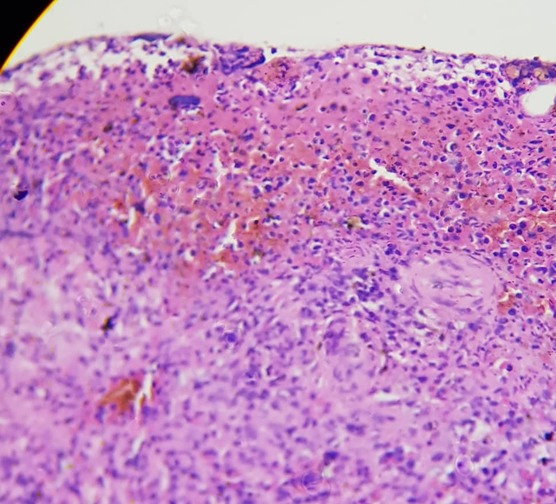
Hématome de la rate

Il s'agissait d'une demoiselle âgée de 25 ans, étudiante, qui nous avait été référée du Centre Hospitalier de Kara pour une insuffisance rénale (IR) oligo-anurique avec une créatinémie à 2210μmol/l, urémie à 46 mmol/l et une anémie sévère à 5g/dl. Il faut noter la prise de médicament traditionnel (Figure 1) par voie orale et endovaginale il y a trois jours dans le but de régulariser son cycle menstruel. Elle ne présentait aucun antécédent personnel et il n'y avait pas de commémoratif familial de néphropathie. Elle était nulligeste nullipare et n'avait signalé aucun traumatisme abdominal notable précédant l'hospitalisation. L'examen clinique à l'entrée avait noté une pression artérielle à 120/80 mmHg, un bon état général, un syndrome anémique clinique, un abdomen légèrement augmenté de volume et des lésions ulcéreuses endovaginales. La première biologie a confirmé l'IR majeure avec 2652 μmol/l de créatinine et 50 mmol/l d'urée, l'ionogramme sanguin était normal, et la NFS notait une anémie sévère à 4,5g/dl et une thrombopénie à 88000 /mm^3^. Devant ce tableau, elle a été hospitalisée dans un service de soins intensifs, et avait été mise en hémodialyse quotidienne avec des séances courtes de 2 heures de temps et avait bénéficié d'une transfusion de 5 poches de culot globulaire. Trois jours après la patiente avait signalé des douleurs abdominales. L'examen de cet abdomen avait montré un abdomen souple, mât, augmenté de volume, sensible dans son ensemble avec un cri de l'ombilic. Le dosage des beta HCG plasmatique était négatif. Deux échographies abdominales réalisées tour à tour avaient montré un liquide anéchogène de grande abondance avec un utérus vide et non gravide. Une ponction exploratrice réalisée avait ramené un liquide hématique non coagulable. Les reins étaient modérément dédifférenciés de taille conservée. On notait ailleurs une hépatomégalie homogène aux pourtours réguliers avec un pancréas normal. Une nouvelle biologie réalisée avait permis de noter la persistance de l'anémie malgré les transfusions itératives avec un taux d'hémoglobine à 6,2g/dl sans schizocytes, l'haptoglobine était normale, les LDH à 600 UI/l, une thrombopénie à 45000/mm^3^, un TP à 31 %, les D-dimères à 800 ng/ml. Il n'y avait pas de troubles phosphocalcique, l'électrophorèse des protides sériques était normale. Une tomodensitométrie abdominale réalisée avait permis d'objectiver un hématome sous capsulaire spontané rompu de la rate (Figure 2). Une prise en charge chirurgicale avec splénectomie totale avait été réalisée après correction des troubles de l'hémostase. L'examen anatomopathologique réalisé confirme qu'il s'agissait bien d'un hématome sous capsulaire avec fracture de la rate sans signe de malignité (Figure 3 et Figure 4). Les suites opératoires avaient été simples. Les séances de dialyse s'étaient poursuivies avec une crise polyurique survenue au cinquième jour post opératoire. Le bilan sanguin réalisé avait montré un taux d'hémoglobine à 10g/dl, les plaquettes à 135000/mm^3^, Un TP à 65 %, une urémie à 11,6mmol/l et une créatininémie à 530μmol/l. A 4 semaines de l'hospitalisation, elle a présenté une récupération partielle de sa fonction rénale qui s'est stabilisée autour de 265μmol/l de créatinine équivalent à une clairance MDRD calculée à 25,52ml/min/1,73m^2^.

## Discussion

À notre connaissance, aucun cas de rupture spontanée de rate secondaire consécutive à une prise de toxique traditionnelle n'a été rapporté. Dans la majorité des cas, une rupture de la rate (RR) est secondaire à un traumatisme abdominal. Les cas de rupture spontanés sont moins fréquents, mais les causes sont nombreuses et parfois iatrogènes (anticoagulant, thrombolytiques, coloscopie, cholangiopancréatographie rétrograde endoscopique) [6]. Dans notre observation, la rupture de rate semble être liée à la CIVD consécutive à la prise de toxique traditionnelle pour plusieurs raisons: l'absence connue de traumatisme abdominal précédent l'hospitalisation, la présence de stigmate de CIVD, et la présentation clinique compatible avec le diagnostic. Le diagnostic de la CIVD a été établi devant une thrombopénie, une élévation du TCA (2,1 fois celui du témoin), ainsi qu'une augmentation des D-dimères. Le dosage du fibrinogène plasmatique n'a pas été réalisé. Diverses étiologies non classiques des CIVD ont été rapportées dont les causes toxiques [7]. Ici, il s'agit de produit toxique traditionnel, qui pourrait entrainer un tableau d'hémolyse intravasculaire. L'association entre la prise de toxique traditionnelle, la CIVD, et l'insuffisance rénale a été rarement rapporté. Bien que certains travaux [8,9] démontrent clairement le rôle de l'hémolyse dans la survenue de l'IRA, ici il parait difficile d'imputer la cause de l'IRA exclusivement au toxique traditionnel. Dans ce contexte, une ponction biopsie rénale transpariétale (PBR) serait le moyen le plus à même de prouver l'imputabilité du toxique. Cet examen n'a pas été réalisé d'une part parce que jugé trop dangereux dans les conditions initiales de l'hospitalisation (présence des troubles de l'hémostase) et d'autres part à cause du refus de consentement de la part de la patiente et des parents. Le toxique retrouvé était fait à base de plantes traditionnelles conditionnées sous forme liquide dans une bouteille (Figure 1). L'analyse toxicologique n'a pas pu être réalisée. Bien que l'intérêt de la pharmacopée traditionnelle soit déjà prouvé dans certaines pathologies, dans nos pays de l'Afrique subsaharienne, il reste un cadre règlementaire basé sur l'analyse toxicologique et pharmacologique à développer.

## Conclusion

La rupture spontanée de la rate est une pathologie rare dont le diagnostic est très difficile en absence de contexte traumatique. L'échographie et le scanner permettent d'orienter le diagnostic. En dehors des causes classiques, la prise de toxique pourrait être une des étiologies et l'insuffisance rénale un mode de découverte.

## Conflits d’intérêts

Les auteurs ne déclarent aucun conflit d’intérêts.
